# Awareness and use of the Rapid Palliative Radiotherapy Program by family physicians in Eastern Ontario: a survey

**DOI:** 10.3747/co.v13i1.184

**Published:** 2006-02

**Authors:** E.J. Fitzgibbon, R. Samant, J. Meng, I.D. Graham

**Affiliations:** * Ottawa Regional Cancer Centre, Ottawa, Ontario; † Ottawa Health Research Institute, Ottawa, Ontario

**Keywords:** Palliative radiotherapy, effectiveness, family physician, awareness, survey

## Abstract

The Ottawa Rapid Palliative Radiotherapy Program (rprp) was established in 1999 with the goal of facilitating access by family physicians to radiotherapy services for patients with advanced symptomatic cancer. Two years later, an audit revealed that of the 148 patients treated by the program, only 19 had been referred by family physicians.

We therefore assessed awareness of the rprp and perceptions of the effectiveness of palliative radiotherapy on the part of family physicians by surveying a random sample of family physicians in Eastern Ontario.

Response rate was 50%. Only 18% of family physicians were aware of the rprp, although 56% had previously referred patients for palliative radiotherapy. Among responders, 80% regularly provided palliative care, and these physicians were much more likely to be aware of and to refer patients for palliative radiotherapy.

Our survey confirms the key role that family physicians play in providing care to patients with advanced cancer. However, significant deficits in family physician awareness of palliative radiotherapy programs and in knowledge of the effectiveness of palliative radiotherapy should be addressed to improve patient care.

## 1. INTRODUCTION

Painful metastatic bone disease is the most common indication for radiotherapy among patients with advanced cancer, accounting for more than 20% of all radiotherapy treatments 1,2. It is estimated that 60% to 80% of patients with advanced cancer will develop bone metastases, with 65% of those patients developing bone pain 3–8. For these patients, local external-beam radiotherapy is considered to be an effective, rapidly acting, palliative intervention 4–6. A systematic review of palliative radiotherapy trials reported that, among patients with painful bone metastases, “complete pain relief” was obtained in 21% to 80% and “partial pain relief” was obtained in 25% to 87%. The median duration of complete pain relief was 12 weeks, regardless of the radiation fractionation schedule used 9.

In 1996, a Canadian symposium on palliative radiotherapy concluded that “a separate palliative radiotherapy clinic, where access is rapid and patients can be assessed, planned, and treated the same day, is an excellent way of handling the special needs of palliative patients” 10. Following this report, the first rapid response radiotherapy program was developed in Toronto, Ontario, to facilitate access to radiotherapy for cancer patients with an estimated life expectancy of less than 6 months 11. Subsequent program evaluations have demonstrated a high degree of support from both patients and referring physicians 12–14.

The Ottawa Rapid Palliative Radiotherapy Program (rprp) was established in 1999 with the specific goal of providing family physicians with rapid access to radiotherapy consultation and treatment for their patients with advanced symptomatic cancer. A retrospective chart audit revealed that 148 patients had been treated by the rprp during the period November 1999 to December 2001. That total included 60 patients referred by 22 family physicians (of which 41 were referred by 5 physicians who were exclusively practicing palliative care) and 88 patients referred by 21 oncologists. Painful metastatic bone disease was the primary reason for referral in 81% of the patients. The primary objective of this survey was to assess awareness of the rprp and barriers to the use palliative radiotherapy among family physicians in eastern Ontario.

## 2. METHODS

### 2.1 Survey Design and Sampling Frame

The survey design was developed by a panel composed of a palliative care physician (EF) a survey methodologist (IG), and two radiation oncologists (JM, RS). The survey questionnaire used the Ottawa Model of Research Use and the Rogers innovation decision process to gather data on factors influencing the adoption of a clinical innovation 15,16. The survey included these sections:

- Respondent characteristics
- Awareness of and perceived accessibility to oncology services at the regional cancer centre
- Factors influencing patient referral for palliative radiotherapy
- Perception of the effectiveness of palliative radiotherapy
- Willingness to attend continuing medical education on radiation oncology

To establish content validity, clarity, and ease of completion, the questionnaire was piloted with 75 family physicians attending an oncology conference. The revised version consisted of 50 questions and required 10–15 minutes to complete.

Family physicians in Eastern Ontario were identified from two lists:

- The Ottawa Regional Cancer Centre list, which included family physicians who had referred patients for cancer treatment from 1991 to 2001
- The list published in the *Canadian Medical Directory* from Southam Information Products

After exclusion of physicians with primary activity in emergency medicine, internal medicine, surgery, and psychiatry, and of those with outdated addresses or telephone numbers, we finalized a list of 997 family physicians. The surveys were administered using a modification of Dillman’s design for the conduct of surveys, with a maximum of four attempts made to contact each physician 17. The investigators jointly signed all letters.

### 2.2 Sample Size

The survey sample size was 400 subjects. This sample was adequate to ensure that a 50% answer to any dichotomous question would be estimated within a confidence interval of 5%. Sample size was adjusted to allow for a minimum expected survey response rate of 50% 18–21.

All surveys received within 12 weeks of the first mail-out were screened to exclude from further analysis those that contained a “yes” answer to either of the questions “I do not practice family medicine” or “I do not have cancer patients in my practice.” Response rates were calculated by dividing the number of completed surveys by the total number of physicians in the sample, minus the surveys excluded as just described 22. Data were analyzed using both the SPSS version 11 (SPSS Inc., Chicago, IL, U.S.A.) and the SAS version 8 (SAS Institute, Cary, NC, U.S.A.) statistical software packages. The Ottawa Hospital Research Ethics Board approved the survey.

Descriptive statistics were used to report answers to survey questions. Unpaired *t-*tests or Pearson chi-square tests were used as appropriate to compare responders with non-responders. Because the survey analysis was descriptive, no adjustment was made for multiple comparisons, however a conservative level of significance (α) of *p* < 0.01 was used to determine the associations that would be reported.

Further statistical analysis included:

- Comparison of the characteristics of family physicians who completed the survey to those who did not
- Tabulation of practice characteristics and awareness and perceptions of palliative radiotherapy
- Use of separate logistic regression models to identify factors independently associated with “awareness” of the rprp and with previous patient referral for palliative radiotherapy

A stepwise modelling format was used to construct multivariate logistic regression models that included variables that met the predetermined selection criteria of a Wald statistic *p*(*z*) < 0.25 in univariate comparisons. No interaction terms were considered in the model-building process.

## 3. RESULTS

Of the 400 randomly selected family physicians, 55 (14%) were excluded for not meeting the survey eligibility criteria. Of the remaining 345 family physicians, 172 (50%) completed the survey, 99 after the first contact, and 73 after a subsequent contact. Physicians who completed the survey differed significantly from those who did not with respect to family practice certification status, practice setting, and hospital admitting privileges (Table I).

As shown in Table II, only 31 responders (18%) were aware of the existence of the rprp, and only 15 (9%) had referred patients to the rprp. However 96 responders (56%) had previously referred patients for palliative radiotherapy outside of the rprp. In addition, 149 of the 172 responders (87%) indicated that they were regularly involved in caring for patients with advanced cancer and 138 (80%) indicated that they regularly provided palliative care to their patients. Of survey responders, 40% had received formal training in palliative care (median: 2 weeks; range: 1–104 weeks). Only 10% had received any teaching in radiation oncology. The responding physicians showed strong support for additional training in both palliative care (81%) and radiation oncology (86%). Small-group workshops were the preferred education format (data not shown).

**Figure 1:**
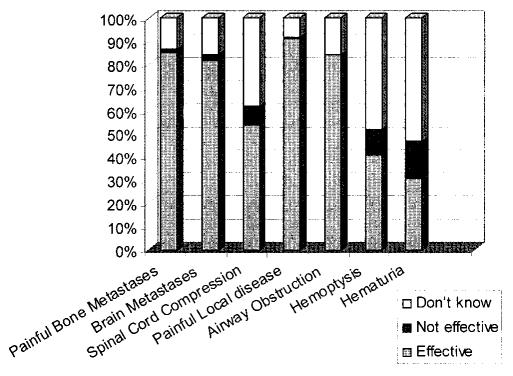
Perception by family physicians of the effectiveness of palliative radiotherapy. For each of indications shown, expert opinion suggests that radiotherapy is “effective” 4,9.

Factors that were significantly associated (*p* < 0.01) with referral of patients for palliative radiotherapy included type of cancer (80%), patient preference (79%), and functional status of the patient (67%). Patient referral was hindered by factors such as long waiting times for radiotherapy (55%) and uncertainty of the benefit of radiotherapy (55%) (Table III). More than 80% of survey responders rated radiotherapy to be an effective intervention in the relief of painful metastatic bone disease, brain metastases, painful local disease, and malignant airway obstruction. However, more than 30% indicated that they did not know if radiotherapy was an effective treatment for other established indications, including malignant spinal cord compression, tumour-associated hematuria, and hemoptysis (Figure 1).

Survey responders who had previously referred patients for palliative radiotherapy differed significantly (*p* < 0.01) from their counterparts who had not made such referrals. These physicians were more often male (73% vs. 27%), more likely to be providing palliative care to their patients (96% vs. 75%), more likely to be practicing outside of urban centres (45% vs. 25%), and more likely to have hospital admitting privileges (62% vs. 40%). They were also more likely to have previously sought advice from a radiation oncologist (73% vs. 29%) and to consider their access to radiation oncologists to be adequate (60% vs. 35%; Table IV).

Multivariate logistic regression revealed that two factors were significantly and independently related to a responder’s “awareness” of the rprp:

- The physician had previously sought advice from a radiation oncologist [odds ratio (or) = 3.13, 95% confidence interval (ci) = 1.15 to 8.53].
- The physician had provided palliative care to patients (or = 3.42, 95% ci = 1.32 to 8.86) (Table V).

The same two factors were even more strongly predictive of the probability of referring a patient for palliative radiotherapy: or = 5.99 (95% ci: 2.89 to 12.42) and or = 5.58 (95% ci: 1.97 to 15.78) respectively.

## 4. DISCUSSION

Among family physicians who responded to our survey, 87% regularly cared for patients with advanced cancer, and 80% provided palliative care. However, only 18% of responders were aware of the existence of the RPRP and just 9% had ever referred patients to the rprp. Despite this, 56% of responders had previously referred patients for palliative radiotherapy outside of the RPRP. Lack of awareness of the rprp is a major factor contributing to the low utilization of the program by family physicians.

The survey revealed that, although only 10% of responders had received formal teaching in radiation oncology, 85% were aware of the effectiveness of radiotherapy in treating painful metastatic bone disease. Most responders (86%) indicated their willingness to attend education sessions in radiation oncology. Interestingly, 55% of responders perceived that the waiting time for patients to receive radiotherapy was a factor that influenced their decision to refer patients for radiotherapy, suggesting that improved access to radiation oncology services via the rprp should improve referral rates.

The fact that only 18% of responders were aware of the rprp indicates that the interventions originally used by the cancer centre to inform family physicians of the establishment of the rprp were not effective. As a first step in correcting this lack of awareness, the survey has identified a subgroup of family physicians who, if they become aware of the program will be likely to use the rprp—that is, the 56% of responders who had previously referred patients for palliative radiotherapy at the cancer centre (Table IV). These family physicians are more likely to be regularly caring for patients with cancer, to be providing palliative care, to have hospital admitting privileges, and to be practicing outside major urban centres. This subgroup of responders matches the profile of the family physician that the rprp intended to assist; logically, this group could be used as a target population for both the design and assessment of interventions to improve awareness of the rprp. Family physicians in this group are widely dispersed throughout Eastern Ontario and could be regarded as a local resource in cancer knowledge for other health care providers in their areas.

Limitations in this study should be acknowledged. Despite repeated contact attempts, the survey response rate was 50%. The survey sampling frame was a composite of two lists, from which eligible physicians may have been excluded. Thus, the survey results may still imprecisely reflect family physician awareness and knowledge of the effectiveness of radiotherapy. Finally, answers to survey questions may have been influenced by social desirability, which may affect our estimate of physician awareness.

## 5. CONCLUSIONS

The results of this survey reveal that, although family physicians play a key role in providing ongoing primary care for patients with advanced cancer, most are unaware of a program that was developed specifically to facilitate family physician access to palliative radiotherapy services. By identifying barriers to the use of the RPRP and suggesting a receptive pilot group of family physicians, the results of the survey can guide the development and delivery of a focused plan to improve knowledge, awareness, and use of palliative radiotherapy services by family physicians. This increased awareness and knowledge will lead ultimately to improved patient care.

## Figures and Tables

**Table I:** Differences in characteristics of physicians according to survey completion

	*Survey completed? [*n *(%)]*		
*Variable*	*Yes*	*No*	p *Value*^a^
CCFP			
Yes	111 (64)	68 (39)	<0.001
No	61 (36)	105 (61)	
Practice setting			
Urban	110 (64)	129 (75)	0.006
Rural	43 (25)	39 (22)	
Mixed	19 (11)	5 (3)	
Hospital admitting privileges			
Yes	89 (52)	72 (42)	0.010
No	83 (48)	101 (58)	
Eligible respondents	172	173	—

a Significant association for reporting set at *p* < 0.01 by chi-square or unpaired *t-*test.

ccfp = certificant of the Canadian College of Family Physicians.

**Table II:** Characteristics of the 172 physicians responding to the survey

*Characteristic*	n *(%)*^a^
Aware of the Rapid Palliative Radiotherapy Program?	
Yes	31 (18)
No	141 (82)
Have referred patients to the rprp?	
Yes	15 (8)
No	157 (92)
Have referred patients for palliative radiotherapy outside of the rprp?	
Yes	96 (56)
No	76 (44)
Number of cancer patients seen in the past month.	
None	6 (4)
1–5	76 (44)
6–10	55 (32)
>10	35 (20)
Frequency of participating in the care of patients with advanced cancer.	
Never	1 (<1)
Rarely	21 (12)
Sometimes	57 (33)
Often	92 (54)
Frequency of providing palliative care.	
Never	5 (3)
Rarely	29 (17)
Sometimes	56 (33)
Often	82 (47)
Previous training in palliative care.	
Yes	69 (40)
No	103 (60)
Training in radiation oncology.	
Yes	17 (10)
No	153 (90)

a The number of physicians who answered each question ranged from 116 to 172.

**Table III:** Factors influencing family physicians to refer patients to palliative radiotherapy

	*Answera(%)*	
	*No*	*Yes*
How much would a family physician’s decision to refer a patient for palliative radiotherapy be influenced by:		
difficulty in contacting a radiation oncologist?	63.8	36.2
uncertainty of the referral process?	73.1	26.9
waiting time for assessment by radiation oncologist?	46.7	53.3
waiting time for radiotherapy?	44.5	55.5
uncertainty of the benefit of radiotherapy?	45.8	54.2
uncertainty of the side effects of radiotherapy?	50.3	49.7
distance a patient lived from the Ottawa Regional Cancer Centre?	56.5	43.5
type of cancer?	20.4	79.6
age of the patient?	57.1	42.9
functional status of patient?	33.1	66.9
anticipated inconvenience to the patient?	50.3	49.7
patient’s preference?	21.5	78.5
life expectancy of patient?	56.8	43.2
What would you consider the minimum life expectancy to be, prior to referring a patient for palliative radiotherapy?	(%)	
<1 month	24.4	
1–3 months	35.9	
4–6 months	21.8	
>6 months	9.0	
Unsure	8.9	

a “No” includes the responses “not at all” and “a little”; “yes” includes the responses “somewhat” and “a lot.”

**Table IV:** Physician factors related to prior referral of patients for palliative radiotherapy

	*Prior Factor referrala(%)*	
*Factor*	*No*	*Yes*
Sex		
Male	29	73
Female	71	27
Regularly provides palliative care?		
Yes	75	96
No	25	4
Urban practice location?		
Yes	75	55
No	25	45
Hospital privileges?		
Yes	40	62
No	60	38
Previously sought advice from radiation oncologist?		
Yes	29	73
No	71	27
Considers access to radiation oncologists adequate?		
Yes	35	60
No	25	32
Never tried	40	8
Considers radiotherapy effective for treating painful bony metastases?		
Yes	92	99
No	8	1

a The number of physicians who provided data on each factor varied from 116 to 172.

**Table V:** Factors related to family physician awareness of the Rapid Palliative Radiotherapy Program a

*Factor*	*Unaware (%) (*n=*141)*	*Aware (%) (*n=*31)*	*Independent predictors [or (95% ci)]*
Previously sought advice b from radiation oncologist?			
Yes	52	81	4.60 (1.78–11.96)
No	48	19	
Provides palliative care c for their patients.			
Yes	77	94	4.26 (0.96–18.80)
No	23	6	
Previous referral of patients for palliative radiotherapy?			
Yes	50	81	4.12 (1.59–10.62)
No	50	19	

	Logit (*aware ofrprp*) = β^0^ + β^1^ + β^2^		
*Variable*	*Regression coefficient (*β*)*	*or (95% ci)*	*Wald* p(z)
0. Intercept	−2.982		
1. Advice	1.142	3.13 (1.15–8.53)	0.026
2. Palliative care	1.228	3.42 (1.32–8.86)	0.012

a Multivariate logistic regression model estimating the probability of a family physician being “aware” of the Rapid Palliative Radiotherapy Program. A significant association (Wald *p(z)* < 0.25 by chi-square) was necessary for a factor to be eligible for entry into logistic model building. Final model:

b Test for model validity: classification = Hosmer and Lemeshow “goodness of fit” (χ^2^ = 2.924, df =6, *p* = 0.82).

c Test for model validity: discrimination = area under the roc curve *c* = 0.69.

or = odds ratio (odds of the outcome occurring for every unit increase in an individual independent variable, controlling for the other variables in the model); ci = confidence interval; intercept = a mathematical constant (no clinical interpretation); roc = receiver operator curve (test for ability of the model to discriminate between variables).
